# Combined therapy of colon carcinomas with an oncolytic adenovirus and valproic acid

**DOI:** 10.18632/oncotarget.22107

**Published:** 2017-10-26

**Authors:** Christian Bressy, Dragomira Majhen, Najat Raddi, Wael Jdey, Gaétan Cornilleau, Léna Zig, Josée Guirouilh-Barbat, Bernard S. Lopez, Olivia Bawa, Paule Opolon, Elodie Grellier, Karim Benihoud

**Affiliations:** ^1^ Vectorologie et Thérapeutiques Anticancéreuses, UMR 8203 CNRS, Université Paris-Sud, Gustave Roussy, Université Paris-Saclay, Villejuif 94805, France; ^2^ Laboratoire Recombinaison-Réparation et Cancer, UMR 8200 CNRS Stabilité Génétique et Oncogenèse, Université Paris-Sud, Gustave Roussy, Université Paris-Saclay, Villejuif 94805, France; ^3^ Unité de pathologie expérimentale de l’IRCIV, Gustave Roussy, Villejuif 94805, France

**Keywords:** colon, polyploidy, oncolytic adenovirus, DNA damage, HDACi

## Abstract

The anti-tumor potential of oncolytic adenoviruses (CRAds) has been demonstrated in preclinical and clinical studies. While these agents failed to eradicate tumors when used as a monotherapy, they may be more effective if combined with conventional treatments such as radiotherapy or chemotherapy. This study seeks to evaluate the combination of a CRAd bearing a ∆24 deletion in E1A with valproic acid (VPA), a histone deacetylase inhibitor, for the treatment of human colon carcinomas. This combination led to a strong inhibition of cell growth both *in vitro* and *in vivo* compared to treatment with CRAd or VPA alone. This effect did not stem from a better CRAd replication and production in the presence of VPA. Inhibition of cell proliferation and cell death were induced by the combined treatment. Moreover, whereas cells treated only with CRAd displayed a polyploidy (> 4N population), this phenotype was increased in cells treated with both CRAd and VPA. In addition, the increase in polyploidy triggered by combined treatment with CRAd and VPA was associated with the enhancement of H2AX phosphorylation (γH2AX), a hallmark of DNA damage, but also with a decrease of several DNA repair proteins. Finally, viral replication (or E1A expression) was shown to play a key role in the observed effects since no enhancement of polyploidy nor increase in γH2AX were found following cell treatment with a replication-deficient Ad and VPA. Taken together, our results suggest that CRAd and VPA could be used in combination for the treatment of colon carcinomas.

## INTRODUCTION

Colorectal cancer (CRC) is the third most common cancer worldwide, with about one million new cases diagnosed and 600,000 deaths per year [1]. Treatment of colon cancer typically involves surgical removal of all or a portion of the colon and may result in serious side effects such as infections, bleeding, and injury to adjacent organs. Surgery may also fail to eliminate metastatic lesions. Chemotherapy and radiotherapy are commonly used as adjuncts to surgery with only minor impact on the overall survival time of the patients [1]. Therefore, new therapies are needed to cure CRC. Recently, virotherapy and treatment with histone deacetylase inhibitors have emerged as useful therapeutic approaches.

Virotherapy uses viruses called oncolytic viruses, which selectively replicate in and kill tumor cells. Several groups have developed oncolytic vectors based on human adenoviruses (Ads) [2, 3] and known as conditionally replicative adenovirus (CRAd). Their tumor selectivity is achieved by controlling viral genome replication with a tumor-specific promoter such as the promoter of telomerase or prostate-specific antigen. Alternatively, selectivity may be obtained by specific deletions within the viral genome. For example, Onyx-015, a type 5 human adenovirus (Ad5) with a deletion in the gene encoding E1B-55kDa protein, replicates selectively in tumors and received regulatory approval in China in the treatment of head and neck cancer [4, 5]. Also, Ads bearing a 24-bp deletion in the E1A region, which prevents interaction with pRb protein, have been shown to replicate in tumor cells that have impairment in the pRb pathway, with minimal replication in post-mitotic cells [6, 7]. Finally, following either direct intratumoral or systemic administration, several pre-clinical and clinical studies have demonstrated the capacity of oncolytic Ads to reduce CRC growth while being well-tolerated [8-11]. Despite these achievements, the efficacy of CRAds is limited by poor transduction of tumor cells, neutralization by the host’s immune system, interaction with blood components, and failure to efficiently spread into tumors [3, 12].

Several strategies have been used to increase the potential of CRAds for the treatment of CRC. For example, CRAds have been armed with a transgene carrying anti-angiogenic functions [13] or with a suicide gene such as cytosine deaminase [14]. Another approach has been to enhance the efficacy of CRAds by combining them with radiotherapy as described for Onyx-015 [5]. More recently, CRAds have been combined with different chemotherapeutic agents (for a review see [15]). For example, the combination of CRAd with 5-fluorouracil [16] or everolimus, an mTOR inhibitor [17], demonstrated enhanced anti-tumor effects in CRC models. Also, in different non-CRC tumor models, histone deacetylase (HDAC) inhibitors have been shown to potentiate CRAds by upregulating Ad5 primary receptors [18] or transgene expression [19].

Histone deacetylases (HDACs) and histone acetyl transferases (HATs) are involved in epigenetic gene regulation. The different classes of HDACs (I, IIa, IIb, III and IV) deacetylate both histones and non-histone proteins. By doing so, they modulate transcription by increasing the level of chromatin compaction, thereby reducing the accessibility of transcription factors to DNA [19]. Interestingly, class I HDACs 1, 2 and 8 have been shown to be overexpressed in colon-derived tumors [20, 21]. HDAC inhibitors (HDACi) are small molecules able to promote histone acetylation by modifying the balance between HDACs and HATs [22]. Their cellular effects include growth arrest through the expression of cyclin-kinase inhibitors, as well as the triggering of apoptotic and cell differentiation pathways, inhibition of angiogenesis, and the activation of anti-tumor immune responses [20]. Among HDACi, valproic acid (valproate, VPA) is a well-established drug used over three decades for the long-term therapy of epilepsy. VPA acts as a specific inhibitor of class I and IIa HDACs and induces proteasomal degradation of HDAC2 [23]. VPA is able to trigger growth arrest and apoptosis *in vitro* in colon carcinomas [24] and reduce adenoma formation in APC^Min^ mice model [21].

In this study, we examine the potential of combining a CRAd and VPA for the treatment of colon carcinoma. We provide evidence that these compounds in combination inhibited CRC growth *in vitro*; and that this effect is not due to an increased CRAd replication but is associated with cell cycle modifications, H2AX phosphorylation, decrease of DNA repair proteins, and polyploidy. Most interestingly, we provide *in vivo* evidence that the combined treatment provoked a stronger reduction of tumor growth compared to single treatments.

## RESULTS

### Reduction of colon carcinoma cell line growth after combined treatment with a CRAd and VPA

**Figure 1:**
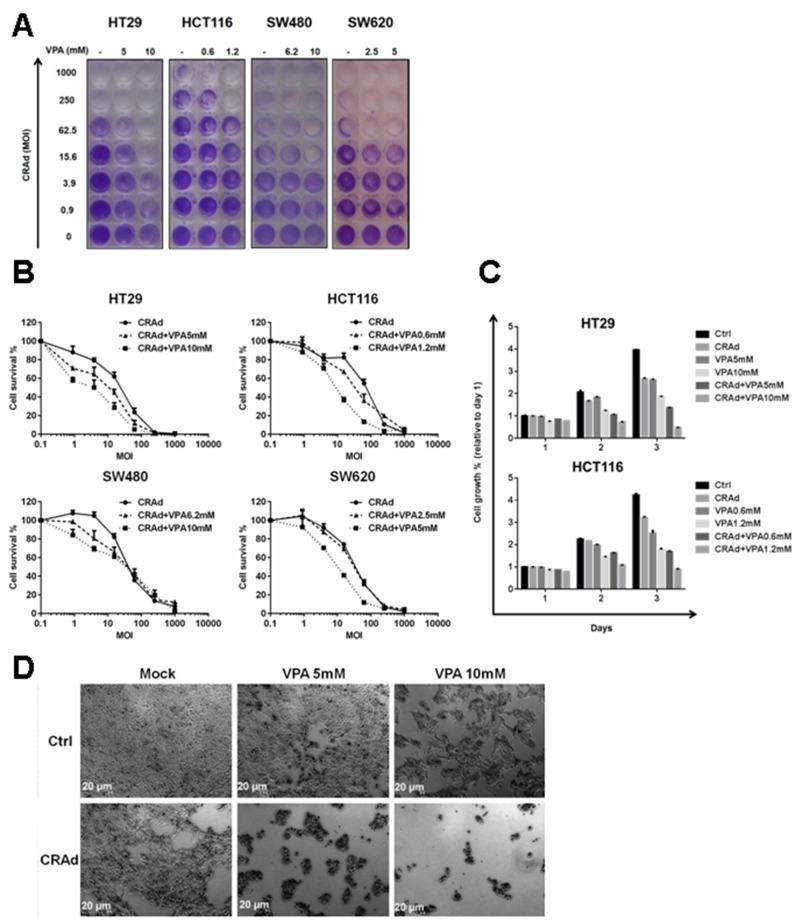
Reduced growth of CRC cell lines after combined treatment with CRAd and VPA CRC cell lines (HT29, HCT116, SW480 and SW620) were infected with different MOI of CRAd (ranging from 0 to 1000 vp/cell) or treated with VPA (IC25 and IC50) or a combination of CRAd and VPA. Cell survival at day 3 was measured by crystal violet **(A)** or MTT **(B)** assays. **(C)** Growth of HT29 and HCT116 was assessed for 3 days by a MTT assay and expressed relative to non-treated cells at day 1. **(D)** After 3 days of treatment, HT29 cells were observed by phase-contrast microscopy. The results are representative of at least two experiments.

In order to improve CRC treatment, we examined whether the combined use of AdE1∆24 (below referred as CRAd) and VPA, a drug already in clinical use, could produce a stronger effect than CRAd or VPA alone. First, using MTT assays we determined VPA doses (Supplementary Table 1) able to reduce the growth of different CRC cell lines (HT29, HCT116, SW480 and SW620). For the continuation of our study we used VPA doses corresponding to IC50 and IC25 for each cell line individually. Then, cells were infected with different MOI of CRAd without or with VPA. After 3 days, a dose-dependant decrease in cell growth for all cell lines, both in crystal violet (Figure 1A) and MTT (Figure 1B) assays, was observed after treatment with CRAd alone, with HCT116 being less sensitive to the virus in comparison to the other cell lines. Compared to the treatment with CRAd or VPA alone, all cell lines treated with both CRAd and VPA displayed a strong reduction in cell growth at MOI ranging from 0.98 up to 62.5 vp/cell. In addition, at these MOI, the reduction in cell growth was more severe with the highest VPA dose (Figure 1B). Specific experiments were performed to assess the synergistic/additive interaction between CRAd and VPA using the Chou-Talalay method [25]. CRAd or/and VPA were added at 0.125 to 2 times their IC50 and cell viability was measured using an MTT assay. Data were used to calculate CI using the Compusyn program. At most tested doses (except higher doses for HCT116), CRAd and VPA reduce cell growth in an additive manner for HT29, HCT116 and SW620. Interestingly, the combination has a synergistic effect in SW480 at different concentrations of the agents (Supplementary Figure 1).

To get insight into the effects of CRAd and VPA combination, we monitored HT29 and HCT116 growth for 3 days after treatment with CRAd, VPA or both (Figure 1C) by MTT assay. A 4-fold increase in cell growth at day 3 was observed in non-treated cells compared to day 1, while cells treated with CRAd or VPA alone showed a 2- to 3-fold increase in cell growth. Interestingly, the combination of CRAd and VPA almost completely inhibited HT29 cell growth (Figure 1C).

On microscopic observation at day 3, while non-treated HT29 cells appeared as a confluent monolayer, a reduced cell number was observed for VPA- or CRAd-only-treated HT29 cells. A dramatic decrease in the number of attached cells was observed after combined treatment with CRAd and VPA (Figure 1D and Supplementary Figure 2) in different cell lines. For all cell lines, the reduction of cell number after treatment with CRAd and VPA was confirmed by cytometry analysis (Supplementary Figure 3).

We also examined the combination of Adβgal, a non-replicative recombinant Ad, and VPA in all colon carcinoma cell lines. In sharp contrast to the results with CRAd and VPA, co-treatment of cells with Adβgal and VPA did not reduce cell survival compared to Adβgal-only-treated cells, suggesting that viral replication or E1 expression is required for the efficacy of CRAd and VPA combination (Supplementary Figure 4A). Furthermore, in contrast to the results observed with CRC cell lines, the co-treatment of prostatic (LNCaP) and renal (786-O) carcinoma-derived cell lines with CRAd and VPA did not reduce cell survival compared to CRAd-only-infected cells (Supplementary Figure 4B).

### Inhibition of cell proliferation and induction of cell death by CRAd and VPA combination

**Figure 2:**
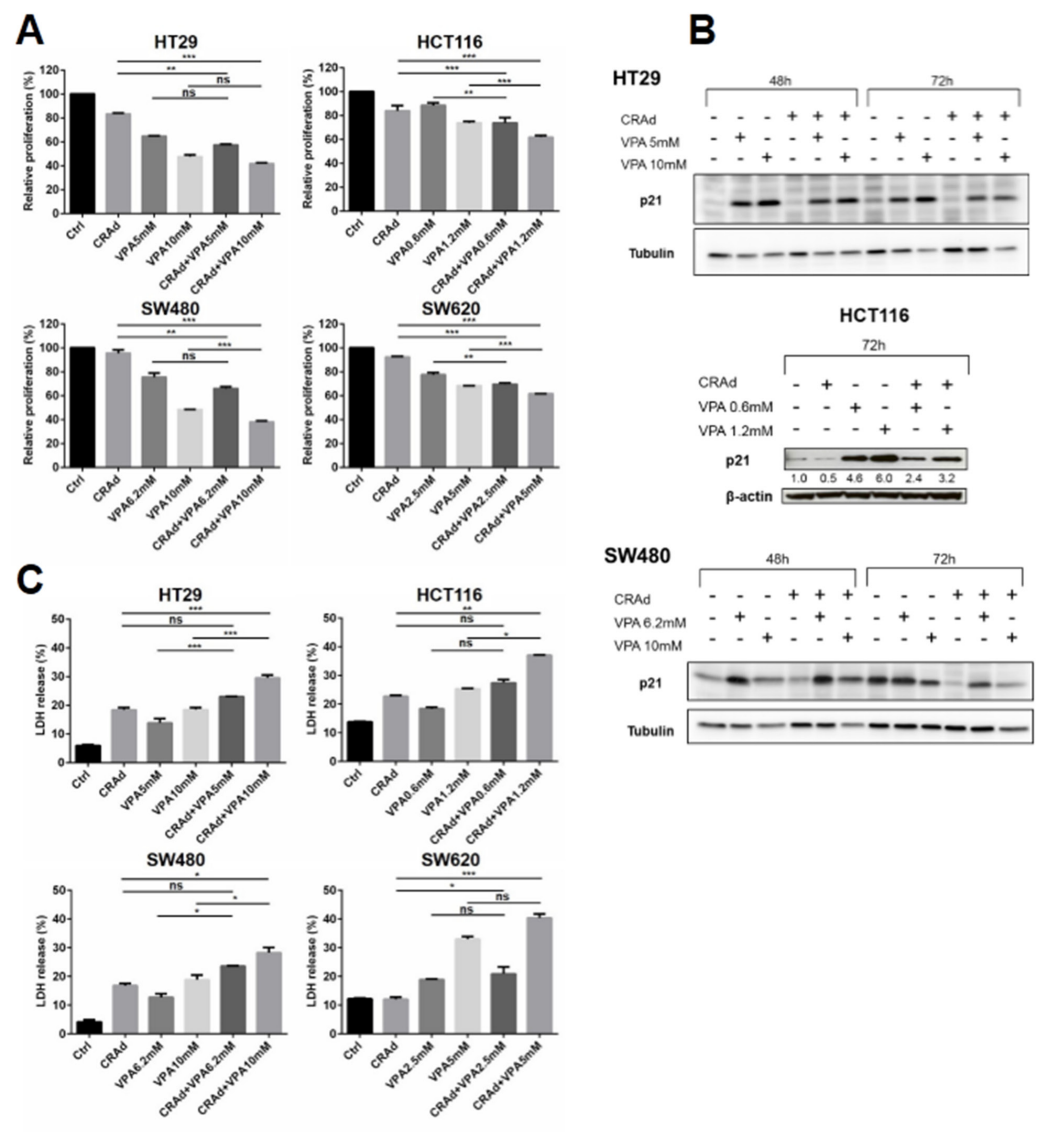
Inhibition of proliferation and increase in cell death of CRC cell lines after combined treatment with CRAd and VPA CRC cell lines (HT29, HCT116, SW480 and SW620) were infected with CRAd (MOI 15.6 vp/cell) with or without VPA (IC25 and IC50) or a combination of CRAd and VPA. **(A)** Relative proliferation index, **(B)** p21 expression in HT29, HCT116 and SW480, **(C)** LDH release were monitored at day 3. Data are from two experiments each performed in duplicate. Means + SD; ns, non significant; ^*^ *P* < 0.05; ^**^ *P* < 0.01; ^***^ *P* < 0.001.

To gain insight into the mechanism underlying the inhibition of cell growth by CRAd and VPA, we first determined the proliferation index of cells 3 days after treatment. Figure 2A shows that the co-treatment significantly reduced proliferation of different CRC cell lines, with the strongest reduction obtained at the highest VPA dose. In order to get insight on p21 status after CRAd and VPA co-treatment we performed western blot analyses on HT29, HCT116 and SW480 cells. In HT29 cells, no p21 expression was found in both Control and CRAd conditions consistent with previous reports [26]. In all three cell lines, CRAd infection led to a reduction of p21 expression compared to control conditions. Interestingly, in these cell lines, a strong dose-dependent induction of p21 by VPA with or without CRAd was found (Figure 2B). This upregulation of p21, a well-known inhibitor of cyclin-dependent kinase may contribute to the inhibition of cell proliferation. Next, we examined whether the co-treatment was able to trigger cell death. Interestingly, in all cell lines, compared to single treatments, CRAd and VPA combined treatment increased cell death as documented by LDH release (Figure 2C). Using annexin V (AV)-binding assay, a strong increase in AV^+^ propidium iodide (PI)^+^ cells was observed in cells treated with CRAd and VPA, with no significant increase in AV^+^PI^-^ cells (Supplementary Figure 5A). Western blot analyses performed on HT29 cell lysates showed no Parp-1 cleavage at day 2 whatever the treatment and low level of Parp-1 cleavage at day 3 after cotreatment with CRAd, VPA (high dose) and CRAd and VPA (high dose). No caspase-3 cleavage was found (Supplementary Figure 5B). These results suggest that apoptosis plays a minimal role in cell death after CRAd and VPA co-treatement.

Taken together, these results demonstrate that CRAd and VPA co-treatment inhibits cell growth through both inhibition of proliferation and induction of cell death.

### Transient delay in viral production after VPA treatment

**Figure 3:**
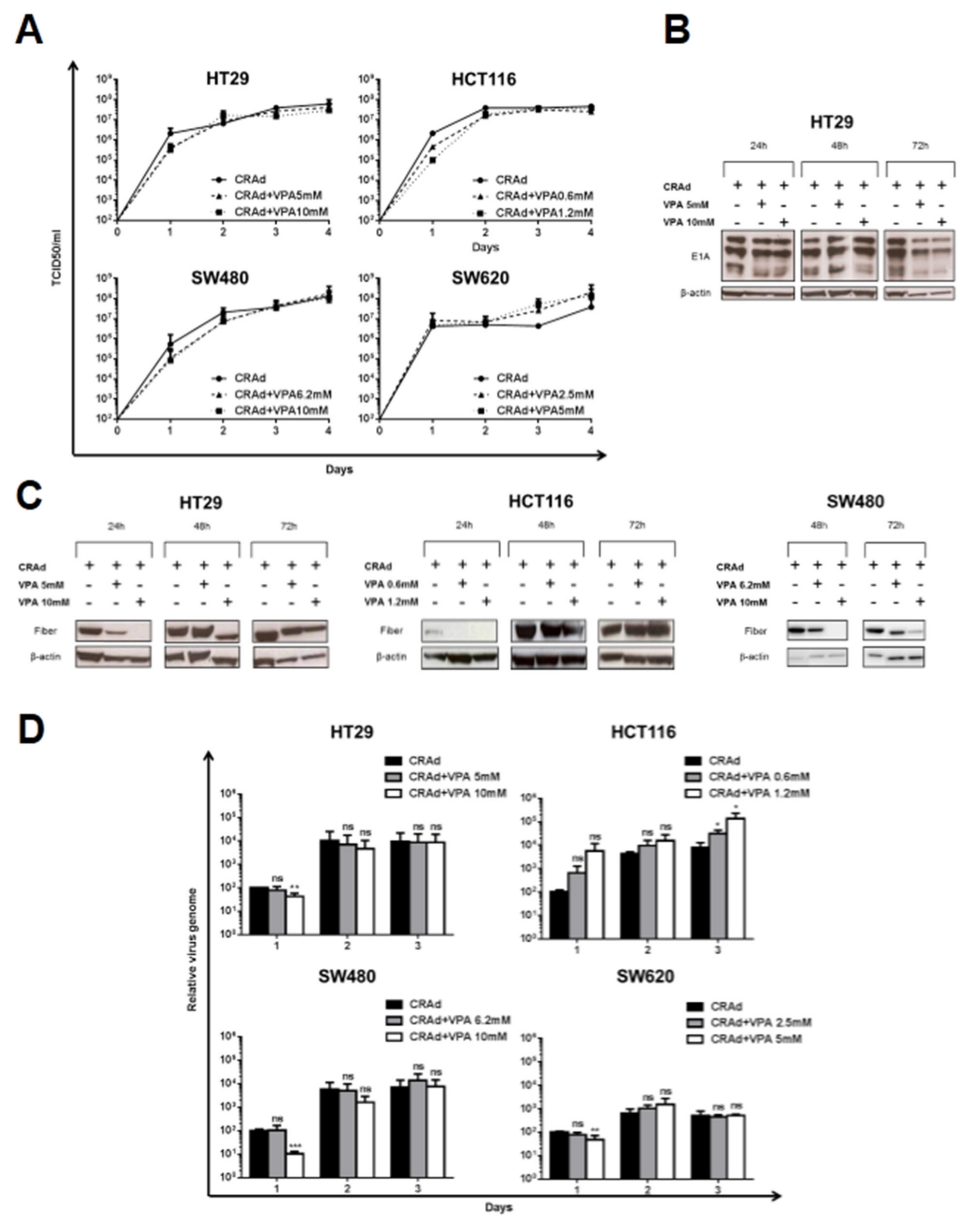
Effect of VPA on CRAd production in CRC cell lines Colon carcinoma cells (HT29, HCT116, SW480 and SW620) were treated with CRAd (MOI 15.6 vp/cell) with or without VPA (IC25 and IC50) for different times. **(A)** Viral production was measured at different time points post infection and expressed as TCID50/ml. Means + SD of 3 to 4 experiments; differences not significant. Expression of E1A early Ad proteins **(B)** and of the fiber late Ad protein **(C)** was detected by western blot. **(D)** Cell-associated viral DNA measured by quantitative PCR is expressed relative to level of viral DNA at day 1 post infection in control-treated infected cells. Means + SD of 2-3 experiments; ns, non significant; ^*^ *P* < 0.05; ^**^ *P* < 0.01; ^***^ *P* < 0.001.

To investigate the molecular mechanism underlying the reduction of cell survival triggered by CRAd and VPA, we examined the ability of VPA to modulate CRAd production. Measurement of infectious virus particles by a TCID50 assay showed a slight, however not significant reduction of viral production at day 1 p.i. in all VPA-treated CRC cell lines (excepted SW620) compared to control-infected cells. Moreover, no statistical difference in viral production was observed at later time points (Figure 3A). Using western blot analyses, a comparable level of E1A proteins was detected in HT29 cells infected with CRAd with or without VPA (Figure 3B) ruling out a role for this drug on modulation of virus infection or early viral gene expression. In sharp contrast, the production of the fiber protein, a late Ad protein, was delayed in the presence of VPA in HT29 (24h and 48h), HCT116 (24h) and SW480 (48h and 72h)-infected cells (Figure 3C). Interestingly, this reduction in the level of a late protein was associated, with the exception of HCT116 cells, with a significant early reduction (day 1) in viral DNA cell content as documented by quantitative PCR (Figure 3D). However, viral genome cell content at later time points was not reduced by VPA treatment in accordance with the comparable viral production observed at these times in VPA-treated and non-VPA-treated cells (Figure 3D and 3A). Altogether, these data demonstrate that the increased cell death triggered by the combination of CRAd and VPA is not associated to an increased viral replication and production.

### Increase in cell ploidy in colon carcinomas following co-treatment with CRAd and VPA

**Figure 4:**
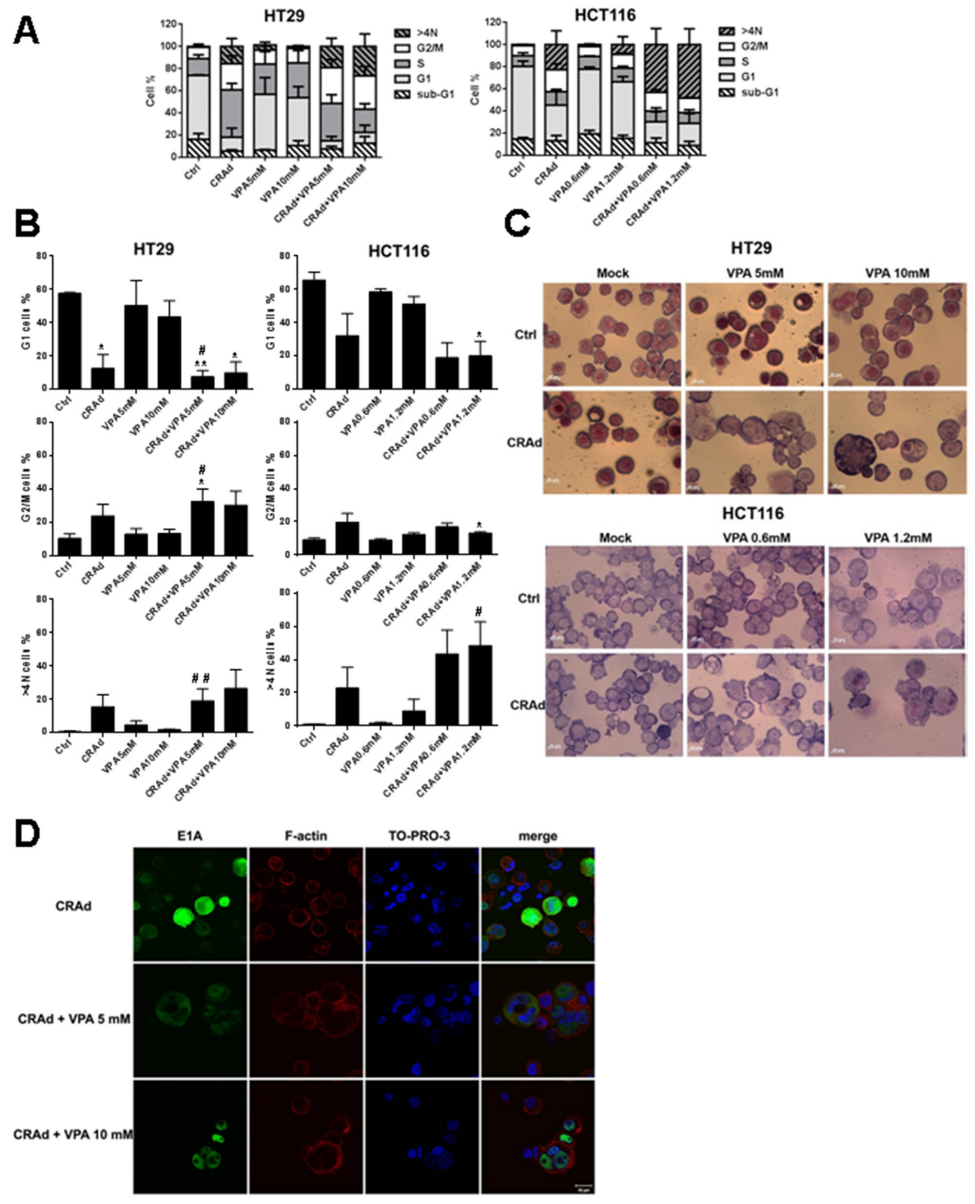
Modification of cell cycle and cell nuclei number after CRC cell line treatment with CRAd and VPA CRC cell lines (HT29, HCT116, SW480, SW620) were untreated (Ctrl) or treated with CRAd (MOI 15.6 vp /cell), VPA (IC25 and IC50), or both. **(A)** Proportion of cells in the different phases of the cell cycle determined by flow cytometry at day 3. **(B)** Similar results as in A displayed to show statistical differences. Means + SEM of 3 experiments; ns, non significant; ^*^ *P* < 0.05, ^**^ *P* < 0.01, ^***^ *P* < 0.001 relative to Ctrl; ^#^ *P* < 0.05 and ^##^ *P* < 0.01 relative to CRAd. **(C)** Cell size and nuclei observed by phase contrast microscopy performed on cells attached via cytospin and stained with Wright-Giemsa at day 3 (scale bar, 20 μm). **(D)** Co-localization of E1A expression and nuclei assessed by confocal microscopy at day 2 in HT29 cells. F-actin was labelled with phalloidin and nuclei with TO-PRO-3; merge is shown (scale bar, 20 μm). The results are representative of two experiments.

To better characterize the effects of the co-treatment on CRC cell lines, we examined their cell cycle distribution following treatment with VPA, CRAd or both. First, we showed that co-treatment with CRAd and VPA, as well as single treatments, did not trigger an increase in the proportion of cells in subG1 phase, thus eliminating a major role of apoptosis in cell death (Figure 4A) in accordance with AV PI assays (Supplementary Figure 5A) and Parp-1/caspase-3 western blot analyses (Supplementary Figure 5B). For both HT29 and HCT116 cell lines, CRAd-infection led to a reduction in G1 phase, and to a trend, even if not significant, to an increase in G2/M and >4N phases. Interestingly, this >4N population was significantly increased after the combined treatment with CRAd and VPA compared to CRAd-only treated cells (Figure 4B). For example, in HCT116 cells treated with CRAd and VPA 1.2mM, the proportion of >4N cells increased up to 48.4% compared to 22.8% after CRAd-only infection. Besides cell cycle analysis, cell size and nuclear morphology were observed by phase-contrast microscopy following Wright-Giemsa staining. Figure 4C shows that some VPA-treated HT29 cells exhibited a small increase in cell and nuclei size compared to untreated cells. Cells infected with CRAd did not show significant change in nuclear morphology. In sharp contrast, HT29 cells exposed to CRAd and VPA co-treatment displayed an irregular nuclear morphology or multiple nuclei (Figure 4C, upper). Similar results were obtained with HCT116 cells submitted to CRAd plus VPA co-treatment (Figure 4C, lower). Cell size was investigated by forward side scatter measurement using flow cytometry. In both HT29 and HCT116 cell lines, CRAd infection triggered a significant increase in cell size (Supplementary Figure 6A). Interestingly, cells co-treated with a recombinant replication-defective Ad (Adβgal) did not display nuclear polyploidy or morphology changes (Supplementary Figure 6B). Using TO-PRO-3 and phalloidin staining to identify nuclei and cell contours respectively, the presence of several nuclei inside one plasma membrane in HT29 cells co-treated with CRAd and VPA was confirmed by confocal microscopy. Such modifications were not observed in infected cells in the absence of VPA (Figure 4D, Supplementary Figure 6B, Supplementary Figure 7A lower) and were restricted to cells expressing E1A, thereby linking this phenotype to virus infection in the presence of VPA. Similar observations were found from the analysis of the HCT116 cellular model (Supplementary Figure 7A). Taken as a whole these results demonstrate that co-treatment with CRAd and VPA triggers cell polyploidy with an increase in nuclei numbers.

### Co-treating cells with CRAd and VPA triggers strong DNA damage and is associated with inhibition of DNA repair proteins

**Figure 5:**
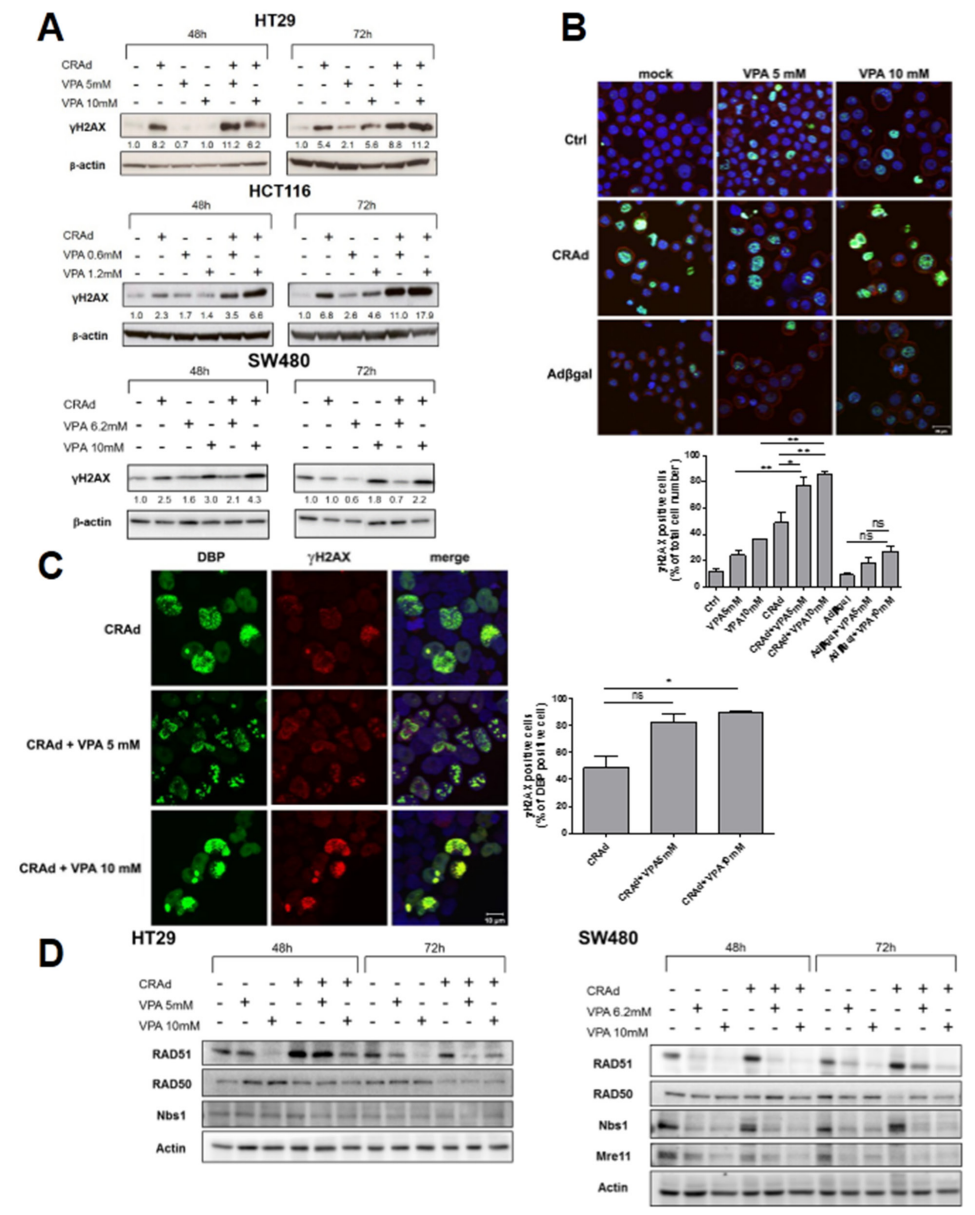
γH2AX induction and inhibition of DNA repair proteins after co-treatment of CRC cell lines with CRAd and VPA CRC cell lines were untreated or treated with CRAd **(A, B, C)** or replication-deficient Adβgal (B) (MOI 15.6vp/cell), VPA (IC25 and IC50), or both. (A) At the indicated time, phosphorylation of H2AX (γH2AX) was measured by western blot. The numbers indicate the level of γH2AX relative to untreated cells. (B) Upper, Expression of γH2AX (green) after 3 days in HT29 cells assessed by confocal microscopy. Actin cytoskeleton and nuclei were respectively stained with phalloidin and TO-PRO-3 (scale bar, 20 μm). Lower, Percentage of γH2AX-positive cells. Means + SD; ^*^ *P* < 0.05 and ^**^ *P* < 0.01. (C) Left, co-localization after 24h in HT29 of adenovirus early DNA binding protein (DBP) (left panel, green) and γH2AX protein (middle panel, red) is shown in merge with TO-PRO-3 stained nuclei (right panel; scale bar, 10 μm). Rigth, Percentage of γH2AX-positive cells among DBP-positive cells. Means + SD; ^*^ *P* < 0.05. The results are representative of two experiments. TO-PRO-3 is represented arbitrary in blue. **(D)** Expression of DNA repair proteins at the indicated time points was measured by western blot.

The increase in polyploidy following CRAd and VPA co-treatment prompted us to examine whether this phenotype could result from a higher incidence of DNA damage. As observed previously by others, VPA [27] and adenovirus [28] independently induce H2AX phosphorylation (Figure 5), a hallmark of DNA double strand breaks (DSB). Figure 5A shows that the co-treatment of HT29, HCT116 and SW480 cells with CRAd and VPA led to a higher γH2AX level compared to cells treated with either CRAd or VPA alone. This increase was detected 2 days after treatment and was even more pronounced after 3 days. In contrast, induction of γH2AX was not found following HT29 co-treatment with replication-deficient Adβgal and VPA (Supplementary Figure 7B), thereby suggesting that Ad replication is required for this phenomenon. Confocal microscopy studies confirmed that after 3 days, the combination of CRAd and VPA but not AdβGal and VPA led to a significant increased percentage of γH2AX-positive (77.0 + 7.0%) cells compared to CRAd-infected (48.8 + 7,9%) or VPA-treated (5mM) non-infected cells (24.3 + 3.0%) (Figure 5B). In addition, co-localization of γH2AX and DNA-binding protein (DBP), a protein expressed during viral replication demonstrated that the highest level of γH2AX at day 1 was induced in infected (DBP-positive) cells only upon VPA treatment (Figure 5C).

To better understand the molecular mechanisms at the origin of the strong H2AX phosphorylation triggered by CRAd and VPA co-treatment, we assessed the expression of different DNA repair proteins by western blot. First, we investigated the expression of the members of Mre11-Rad50-Nbs1 (MRN) complex, a DSB sensor that is required for DNA repair by recombination. Figure 5D showed that CRAd led to a reduction of Rad50 at day 3 in both HT29 and SW480 cell lines, a reduction of Mre11 at day 3 in SW480 cells but has no activity on Nbs1 in both cell lines. VPA led to a strong reduction of Nbs1 and Mre11 levels in SW480 as soon as day 2 post-treatment but has no influence on levels of these proteins in HT29 (Figure 5D and data not shown). We also investigated whether the levels of proteins belonging to non-homologous end joigning (NHEJ) or homologous recombination (HR) DNA repair pathways could be modified by CRAd, VPA or both. Interestingly, after 3 days of infection with CRAd but not after treatment with VPA alone, both HT29 and SW480 displayed a reduced level of ligase IV, a key enzyme of the NHEJ process (Supplementary Figure 8). In addition, VPA but not CRAd triggered a reduction of Rad51, a key enzyme of the HR process, this reduction being more prominent in SW480 cells (Figure 5). These results demonstrate that CRAd and VPA independently down-regulate several key proteins of DNA repairs pathways. The down-regulation of several proteins observed in SW480 could explain the synergistic effect of the combination on the survival of this cell line (Supplementary Figure 1).

Since HDACi are potent reactive oxygen species (ROS) inducers [29, 30] and ROS could mediate DNA damage, we measured the level of ROS production in HT29 cells treated with CRAd, VPA and both using a fluorescent probe. The results indicated that the combined treatment elicits a strong production of ROS compared to single treatments (Supplementary Figure 7C). Altogether, these results suggest that the production of ROS could be at the origin of the increase of γH2AX.

### Co-treatment with CRAd and VPA reduces tumor growth

**Figure 6:**
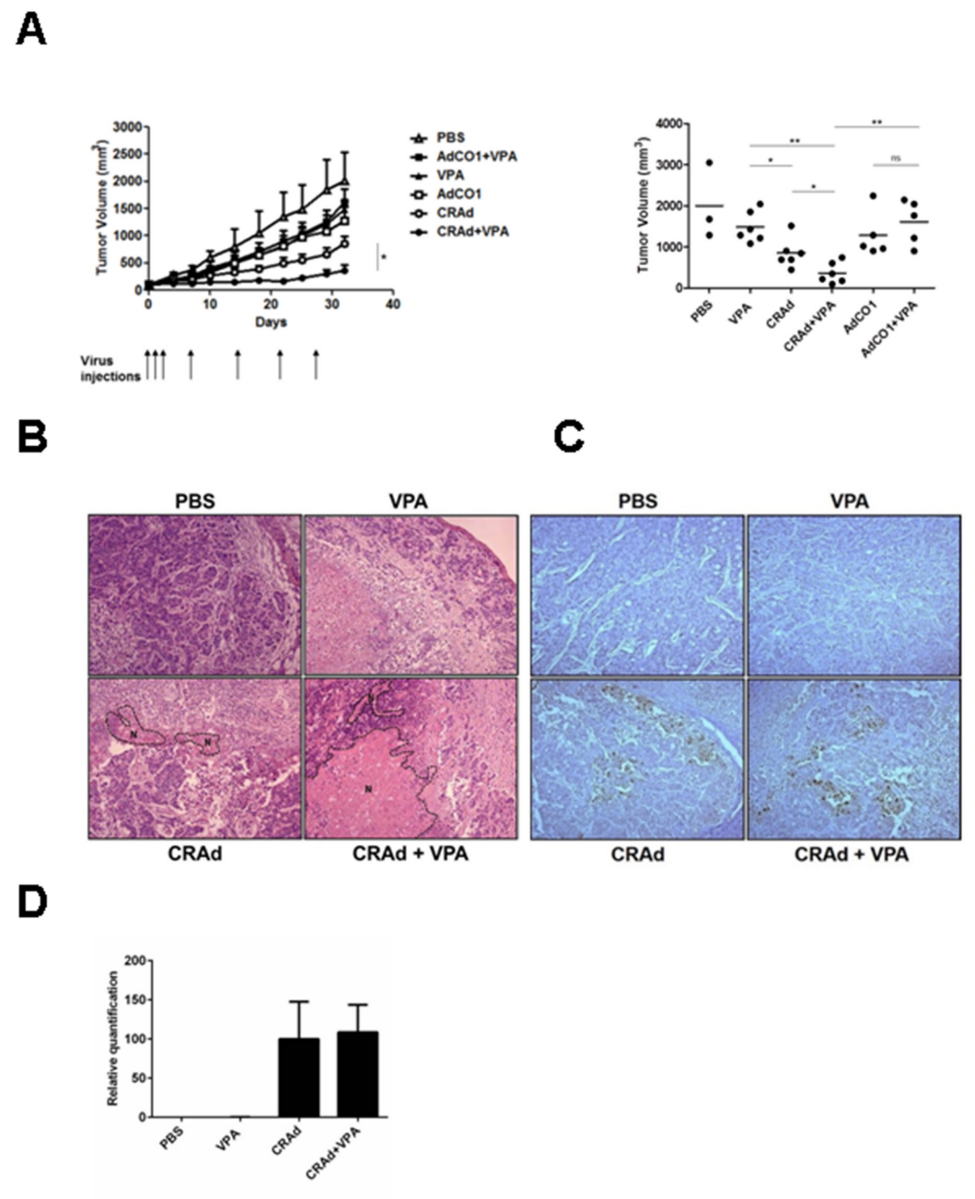
Reduction of tumor growth following the treatment of colon carcinoma tumors with CRAd and VPA Mice bearing HT29-derived xenografts were injected intraperitoneally with daily injection of VPA (300 mg/kg) or PBS and at the indicated time points (arrows) injected intratumorally with virus (CRAd or non-replicative AdCO1) or PBS. **(A)** Kinetics of tumor growth showing means + SEM (left) and tumor volumes at day 32 with dots and bars representing results of individual mice (n = 3 to 6) and means, respectively (right). At day 10 of the treatment **(B)** HES staining of tumor sections (magnification × 100) showing areas of necrosis (N) surrounded by dotted lines, **(C)** detection of hexon protein in tumor sections by immuno-histochemistry (magnification × 100), and **(D)** measurement of viral genome level by Q-PCR. ^*^ *P* <0.05 and ^**^ *P* <0.01.

Having characterized the effects of the combined treatment with CRAd and VPA on CRC cell lines *in vitro*, we evaluated its therapeutic potential using nude mice bearing HT29 xenografts. First, we determined that a daily dose of VPA of 300mg/kg led to a slight reduction of tumor growth, even if not statistically significant (Supplementary Figure 9). This dose was used in subsequent experiments to assess the ability of VPA to modify tumor growth when combined with CRAd. Of note, this dose corresponds after extrapolation based on body surface area [31] to the human equivalent dose of 24.3mg/kg far below the maximal tolerated dose in humans of 60 mg/kg/day [32]. Mice were injected subcutaneously with HT29 cells, then after 12 days were injected intraperitoneally with PBS or VPA and intratumorally with either CRAd, a non-replicative virus (AdCO1), or PBS. Compared to VPA- and CRAd-injected mice, mice injected with CRAd and VPA displayed a significant reduction of tumor growth (P < 0.01 and P < 0.05, respectively). Most interestingly, mice injected with CRAd and VPA displayed significantly reduced tumors compared to mice treated with a replication-deficient AdCO1 and VPA (Figure 6A, P < 0.01). This effect is associated with an increase in the necrotic areas in tumors retrieved from CRAd-plus-VPA-injected animals compared to CRAd-only injected animals (Figure 6B). Of note, this combined treatment was well tolerated since mice did not exhibit any significant weight loss (data not shown).

In order to better understand the significant anti-tumor efficacy observed between CRAd-plus-VPA versus CRAd-only injected mice, we examined the level of viral protein expression and viral replication in both groups. After 10 days of treatment, expression of viral hexon protein assessed in tumor sections by immunohistochemistry was comparable in both groups (Figure 6C). In addition, no difference was observed in viral production as documented by the measurement of viral genome (Figure 6D) and infectious particles (data not shown) by quantitative PCR and TCID50 assays, respectively. Altogether, our results indicate that combining CRAd with VPA led to a strong anti-tumor effect that is not associated with a better viral replication.

## DISCUSSION

Oncolytic Ads used as a monotherapy do not replicate sufficiently in the tumors to have a significant therapeutic effect. Therefore, combining oncolytic Ads with other treatments is mandatory to achieve their full potential. In the present study, using different CRC cell lines, we demonstrated that combining CRAd with the HDACi VPA led to a poorer cancer cell survival compared to CRAd or VPA alone, both *in vitro* (Figures 1 and 2) and *in vivo* (Figure 6). Interestingly, such an effect was not found with the combination of a replication-deficient Ad and VPA. In addition, this effect was not observed with cell lines of renal or prostatic origin that are known to express low levels of CAR receptor [33, 34], thus suggesting it was linked to the efficient CRC cell line transduction by CRAd.

Other teams have investigated the potential of a combined treatment with an oncolytic Ad and HDACi with some discrepancies in the results. Thus, several teams reported an increased viral replication in different types of tumors due to a HDACi-mediated upregulation of CAR expression and better virus entry [35-38]. In contrast, one study reported that VPA inhibit adenovirus replication in both prostatic and colon carcinomas but experiments were conducted using a wild-type and not an oncolytic Ad [39]. Finally, a recent study reported no modification of CRAd replication after glioma cell treatment with VPA [40].

Taking into account these studies, we first measured viral replication and viral protein expression in different CRC cell lines to get insight into the therapeutic effect of the co-treatment with CRAd and VPA. Whereas a reduction in the level of viral genome and late protein (fiber) production was observed at day 1 post-infection in cells treated with VPA, viral particle production at later time points remained unaffected (Figure 3). In addition, the levels of viral replication and late protein (hexon) expression were comparable in CRAd-injected tumors of mice treated with or without VPA (Figure 6). Taken as a whole, the therapeutic effect mediated by the combination of CRAd and VPA does not stem from a better viral replication.

The efficacy of the combination of CRAd and VPA was shown using both MTT and crystal violet assays (Figure 1). Since the two methods do not discriminate between cell death and reduction in cell proliferation, the effect of the co-treatment on CRC cell lines was investigated using more accurate methods. First, for all cell lines, we reported a reduction of cell number after 3 days of treatment with VPA or CRAd that was even more pronounced in co-treated cells (Supplementary Figure 3). The VPA-induced reduction of cell number was already reported for different types of tumor cells, including a CRC cell line [41]. The fact that an increase in p21 expression was observed following cell treatment with VPA or CRAd and VPA in HT29, HCT116 and SW480 cells provides a molecular basis to the inhibition of proliferation observed in these cells co-treated with VPA alone or combined with CRAd. In addition, since p21 was previously shown to bind pro-caspase-3 and inhibit its cleavage [42], the expression of p21 may account for the lack of apoptosis in these cell lines. Of note, no senescence associated-β-galactosidase (SAβGal) activity was found following CRAd and VPA cotreatment ruling out that the inhibition of proliferation occurs through a p21-mediated process of senescence [43]. Second, with the exception of SW620, a slight increase in cell death was found in cells co-treated with CRAd and VPA, as documented by LDH release. No increases were found in the subG1 population (Figure 4) and Parp-1 or caspase-3 cleavage (Supplementary Figure 5B), thereby excluding a massive apoptotic cell death. Besides p21 upregulation, the absence of apoptosis in our cell lines might be due to the mutated status of *APC* leading to increased survivin level and resistance to apoptosis as described previously following treatment with VPA of different CRC cell lines [44].

During our analysis of the cell cycle, we observed the emergence of a > 4N population in CRAd-infected cells as reported by others [45, 46]. In contrast, VPA was unable to trigger significant occurrence of the > 4N population in accordance with its capacity to inhibit cell proliferation [41]. Interestingly, cells co-treated with CRAd and VPA displayed an increase in the > 4N population (Figure 4B) as well as a larger size and a higher nuclear content (Figure 4C). Such an increase in > 4N population was also documented by others after cell treatment with oncolytic Ad and an aurora B inhibitor [45] or paclitaxel, a drug that stabilizes microtubules [47]. In our studies, using confocal analyses, we linked this > 4N population to the occurrence of polyploidy in CRAd-infected cells only after treatment with VPA. This polyploidy in oncolytic Ad-infected cells is more likely the consequence of VPA-mediated inhibition of key steps in cell division, as observed with other chemotherapies [45, 47].

VPA and CRAd are known to inhibit DNA repair independently. Indeed, VPA is able to trigger, in a dose-dependent manner, the phosphorylation of histone H2AX, a hallmark of DSBs [27]. Recently, VPA was shown to block DSB repair through an autophagy-dependent downregulation of CtIP (Sae2) and Exo1, both of which are involved in sensing DSB [48]. Adenovirus infection induces γH2AX [28] and targets different proteins involved in DNA repair (MRE11, ligase IV and DNA-PK), leading to their inactivation/relocalization/degradation [49, 50]. Our results confirmed strong H2AX phosphorylation induced by CRAd and to a minor extent by VPA, but also highlighted a stronger γH2AX level following cell co-treatment with CRAd and VPA (Figure 5A and 5B). Interestingly, such an increase in γH2AX was not found following cell co-treatment with a replication-deficient Ad and VPA (Supplementary Figure 7B), suggesting a role of viral replication (or E1A expression) in accordance with a previous report [28]. This observation together with the lack of polyploidy observed in VPA-treated cells infected with a replication-deficient Ad (Supplementary Figure 6B) suggest that an increased level of DSB in cells co-treated with CRAd and VPA underlies the genetic instability and increased polyploidy. This polyploidy could be responsible for the inhibition of cell proliferation as observed by others [51].

To understand the origin of DSB, we analysed the influence of CRAd and VPA on the expression of DNA repair proteins in two CRC cell lines. Interestingly, CRAd alone reduces the level of Rad50 in both HT29 and SW480 cell lines and inhibits Mre11 protein level in SW480 (Figure 5D). Our results are in accordance with a previous study showing degradation of Rad50 and Mre11 by Ad early proteins E1B55kDa and E4orf6 [49]. However, in contrast to this study, Nbs1 level in our cell model was not modified by CRAd. In addition, we also observed that CRAd-infected cells display a reduced level of ligase IV, a critical component of NHEJ pathway (Supplementary Figure 8). This could be linked to a E1B55kDa and E4orf6-mediated degradation process as described previously [52]. Besides CRAd-induced down-regulation of DNA repair proteins, we reported that VPA alone led to a dramatic reduction of Rad51 in both HT29 and SW480 and to a decrease of Nbs1 and Mre11 levels in SW480 cells but not in HT29 cells (Figure 5D and data not shown). Modulation of protein levels by VPA was reported earlier for Rad51 [53, 54] but neither for Nbs1 nor for Mre11. Thus, CRAd and VPA act independently to reduce different proteins of DNA repair. They could on also act together to modulate Mre11 as suggested by lower Mre11 levels in CRAd and VPA treated cells compared to single treatments (Figure 5D). As a result, the reduction of Mre11 levels could impair the correct telomere maintenance leading to their fusion [55-58] and the observed polyploidy (Figure 4). Of note, this polyploidy is not related to microsatellite stability (MSS) or instability (MSI) phenotype. Indeed, HT29, SW480 and SW620 displaying a MSS phenotype present a polyploidy comparable to HCT116 having a MSI phenotype [59]. Finally, our observation of a stronger ROS production after CRAd and VPA cotreatment (Supplementary Figure 7C) suggests that ROS by their ability to trigger DNA damage may participate to the strong induction of γH2AX.

In conclusion, our results demonstrate that the combination of CRAd and VPA leads to a strong reduction of colon carcinoma growth both *in vitro* and *in vivo*. This effect requires CRAd replication since such effect was not found both *in vitro* and *in vivo* using a replication-deficient Ad and VPA. The inhibition of cell proliferation and the induction of cell death are the main mechanisms of action of CRAd-VPA combination. In our *in vivo* experiments, CRAd and VPA were injected intraperitonally and intratumorally, respectively. Other ways of administration of CRAd such as direct injection into orthotopic colon tumors or intravenous injection should help to ascertain the potential of CRAd-VPA combined therapy for the cure of colon carcinomas. The development of such co-treatment should be facilitated by the feedback from previous clinical trials targeting cancer and testing VPA or CRAd.

## MATERIALS AND METHODS

### Cell lines

Colon-(HT29, HCT116, SW480 and SW620), prostatic-(LNCaP) and renal (786-O)-derived carcinomas were obtained from American Type Culture Collection (ATCC) and maintained in media recommended by ATCC. Of note, whereas HT29, SW480 and SW620 present a microsatellite stability (MSS) phenotype, HCT116 displays a microsatellite instability (MSI) phenotype [59].

### Adenovirus

Adβgal, a lacZ recombinant Ad with E1 and E3 regions deleted [60], and AdCO1, which expresses no transgene [61] were used in these studies. AdE1∆24 was derived from Adβgal using the following procedure: First, the E1Δ24 gene from pXC1-Δ24 carrying a 24 bp deletion removing the pRb-binding CR2 domain of E1A (kindly provided by Dr VW van Beusechem) was cloned into pXL3048. Recombination in *E. coli* of pXL3048-E1∆24 and the Adβgal genome enabled us to obtain the AdE1∆24 genome. All viruses were produced and purified as described previously [62]. Titers were measured by spectrophotometry (1 OD_260_ = 1.1× 10^12^ vp/ml).

### Cell viability and Chou-Talalay analysis

Cells (10^4^ to 5 × 10^4^) were plated on 96-well plates and treated with different multiplicity of infection (MOI) of AdE1∆24 (referred below as CRAd) and/or different doses of VPA (Sigma-Aldrich), as defined in Supplementary Table 1. After 3 days, the medium was removed and cells were stained with crystal violet 0.2% for 15 min. Then, plates were washed and dried before performing macroscopic observation. Alternatively, cell viability was measured at different time points using an MTT assay. The results (mean ± SD) were expressed relative to VPA-matched non-infected cells or relative to cell control at day 1 for the kinetic experiment.

For Chou-Talalay analysis, fixed ratios of the IC50 value of VPA and/or CRAd where used to treat the cells either individually or in combination. Combined dose-response curves were fitted to Chou-Talalay lines [25]. Combination indexes (CI) were determined using CompuSyn software (ComboSyn Inc., Paramus, NJ). CI < 1.2, 0.8 < CI < 1.2 and CI > 1.2 indicate synergistic, additive and antagonistic interactions, respectively.

### Cell proliferation and death

For carboxyfluorescein succinimidyl ester (CFSE) staining, cells were harvested and loaded with 5 μM CFSE for 10 min at room temperature. After washing with PBS, cells (5 × 10^5^) were seeded in 6-well plates and exposed the next day to different conditions. The proliferation index corresponds to the ratio of CFSE mean fluorescence intensity of cells before treatment to mean fluorescence intensity after 3 days of treatment. The proliferation index was expressed relative to the control-treated cells.

For the specific measurement of cell death, cells (5 × 10^5^) were plated on 6-well plates and treated as described above. Then, supernatants were collected and lactate dehydrogenase (LDH) activity was measured using *in vitro* toxicology assay kit (Sigma Aldrich). The results were expressed relative to total LDH level obtained with cells treated with identical conditions and permeabilized with triton 0.1%.

### ROS production

Cells seeded in 6-well plates, (5 × 10^5^ cells/well), were treated with AdE1∆24 (MOI 15.6 vp/cell), with or without VPA (low and high doses). Cells were harvested, washed with PBS and incubated with Dihydroethidium (0.5μM). After 20min, cells were analyzed by cytometry using FACscan cytometer (Beckson Dickinson) and CellQuest software (Beckton Dickinson).

### *In vitro* and *in vivo* virus production

Cells seeded in 6-well plates, (5 × 10^5^ cells/well) were infected with AdE1∆24 (MOI 15.6vp/cell), with or without VPA, in adequate medium supplemented with 2% FBS. After one hour, 3 ml of growth medium with or without VPA was added. Then, at different time points post-infection (p.i.), cells were scrapped and, cells and medium were harvested. After freeze-thaw cycles, the number of infectious particles was determined by TCID50 assay on 293A cells (Invitrogen). The results are expressed as TCID50/ml.

For quantification of the viral genome, cells were harvested and, after centrifugation and washing, total DNA was extracted using Nucleospin Tissue kit according to the manufacturer instructions (Macherey-Nagel, Hoerdt, France). The viral genome was assessed using primers for the viral gene hexon: forward (5′-CTTACCCCCAACGAGTTTGA-3′) and reverse (5′GGAGTACATGCGGTCCTTGT-3′). As an internal positive control, eukaryotic 18S rRNA was amplified using the following primers: forward (5′-CGTTCAGCCACCCGAGAT-3′) and reverse (5′-AACCTGCGGAAGGATCATTA-3′). Amplification was monitored in duplicate on a StepOne Real-Time PCR system (Applied Biosystems, Perkin-Elmer) using SYBR Green reagent (Applied Biosystems, Perkin-Elmer) according to the manufacturer instructions, under the following conditions: 2 min incubation at 50°C, 10 min at 95°C, followed by 40 cycles of repeated incubations at 95C° for 15 s and 60°C for 1 min. Viral genome levels in cells were analyzed by the comparative ΔΔCt method and expressed relative to the Ad-genome level measured in cells infected with AdE1Δ24 alone.

### Western blot

Cells seeded in 6-well plates, (5 × 10^5^ cells/well), were treated with AdE1∆24 (MOI 15.6 vp/cell), with or without VPA (low and high doses). At indicated time points post infection (p.i.), cells were harvested and protein extracts were prepared in RIPA buffer (10mM Tris base pH 8.8, NaCl 150mM, EDTA 1mM, NP-40 1%, deoxycholic acid 1%) supplemented with a mini EDTA-free protease inhibitor cocktail tablet (Roche). Protein extracts (25 or 40 μg) were run in NuPAGE (Invitrogen, France), 9% SDS-PAGE gels or 3-8% Tris Acetate precast gels (Biorad, Hercules, CA, USA), transferred onto a nitrocellulose membrane, and probed with anti-fiber (AB4 clone 4D2, Thermoscientific), anti-E1A (sc-430, Santa-Cruz), anti-phospho-histone H2AX (anti-γH2AX (ser 139), clone JBW301, Millipore), anti-p21 (OP-64, Calbiochem or Santa Cruz Biotechnology, Dallas TX, USA), anti-RAD51 (Merck Millipore, Darmstadt, Germany), anti-Ligase IV (Genetex, Taiwan, R.O.C), anti-Nbs1, anti-DNA-PK and anti-Ku70 (Thermofisher scientific, Waltham, MA, USA), anti-RAD50 and anti-Vinculin (Abcam, Cambridge UK), anti-p21 (Santa Cruz biotechnology), anti-αTubulin and anti-βactin antibodies (Clone AC-15, Sigma-Aldrich), followed by HRP-conjugated secondary antibody. When required, protein extracts were sonicated before separation on PAGE. The western blot results were quantified by Image-J software and expressed as arbitrary units relative to untreated cells. Immunoreactivity was visualized using an enhanced chemoluminescence detection kit (ECL, Pierce).

### Cell cycle

Cells seeded in 6-well plates, (5 × 10^5^ cells/well), were untreated or treated with CRAd (MOI 15.6 vp/cell) with or without VPA (low and high dose) for 72 hours. Then, cells were harvested, centrifuged, and resuspended in sodium citrate buffer containing Triton X-100 0.1%, RNAse 100μg/ml, and propidium iodide 50μg/ml. Cell cycle distribution was determined by flow cytometry analysis performed on Facscalibur (Beckman).

### Morphologic evaluation with Wright–Giemsa dyeing

Cells seeded in 6-well plates, (5 × 10^5^ cells/well), were untreated or treated with virus (CRAd or Adβgal, MOI 15.6 vp/cell) with or without VPA (low and high dose). After 72h, cells were harvested, attached to slides *via* cytospin, fixed with Wright stain (WS16, Sigma) and stained for 15 minutes in Giemsa solution (GS500, Sigma). After washing, dry slides were mounted with a coverslip. Cells and nuclei observations were performed using phase-contrast microscopy.

### Confocal microscopy

Cells seeded in 6-well plates, (5 × 10^5^ cells/well), were untreated or treated with CRAd or Adβgal (MOI 15.6 vp/cell) with or without VPA (low and high dose). After two days, adherent cells were detached with TrypLE™ Express (Invitrogen) and collected together with non-adherent cells and attached on slides by using cytospin centrifuge, 500 rpm during 5 min. Cells grown on a cover glass were washed with PBS and fixed with 2% paraformaldehyde for 12 min. After permeabilization with Triton X-100 0.5% for 10 min, cells were blocked with PBS containing 3% BSA for 30 min. Samples were exposed overnight to primary antibodies (anti-E1A sc-430; anti-DBP, a gift from Dr D.F. Klessig; anti-γH2AX) in PBS-BSA followed by Alexa Fluor-conjugated secondary antibody labeling for 4h. Nuclei were contrast stained with TO-PRO-3 and Alexa Fluor 555-labeled phalloidin (Sigma) was used to selectively label F-actin. Slides were mounted in mounting media and observed using the Zeiss LSM 510 confocal microscope.

### *In vivo* experiments

For *in vivo* studies, all animal experiments were approved by the IGR Institutional Animal Care and Use Committee. HT29 cells (10^7^ cells) in 100μl of PBS were injected subcutaneously into athymic NU/NU female mice housed at the Institut Gustave Roussy. When tumors reached 70-100 mm^3^, mice were injected intratumorally with virus (AdE1∆24 or AdCO1) or PBS for three consecutive days every week for 4 weeks. In parallel and beginning at day 4, mice received intraperitoneal injections of VPA (300mg/kg) or PBS in a volume of 200μl five consecutive days per week until the end of the protocol. Tumor growth was measured twice per week and weight five days per week for 4 weeks. In some experiments, mice were sacrificed, tumors were harvested and total DNA was extracted using Nucleospin Tissue kit. Viral genome content was determined by real-time quantitative PCR as described above.

### Histology and immunohistology

Tumors were excised, fixed in FineFix (Milestone), and embedded in paraffin. Sections (4 μm) were stained with hematoxylin-eosin-safranin (HES). Hexon protein was detected using a polyclonal anti-adenovirus hexon protein (AB1056, Chemikon, CA) and a biotinylated rabbit anti-goat immunoglobulin antibody conjugated to streptavidin-horseradish peroxidase (DAKO, France); slides were counterstained with hematoxylin.

### Statistical analyses

For *in vitro* experiments a two-tailed paired t test was conducted using Graphpad Prism. For *in vivo* experiments, a Mann–Whitney test, recommended for groups fewer than 30 mice, was conducted. Differences were considered significant when *P < 0.05*.

## SUPPLEMENTARY MATERIALS FIGURES AND TABLE


