# Supplementary material for: Combined therapy of colon carcinomas with an oncolytic adenovirus and valproic acid
Source: Oncotarget. 2017 Oct 26;8(57):97344–60. doi: 10.18632/oncotarget.22107 (PMC5722567; doi:10.18632/oncotarget.22107)
Supplement: Supplementary file 1 [file oncotarget-08-97344-s001.pdf]

Combined therapy of colon carcinomas with an oncolytic adenovirus and valproic acid

SUPPLEMENTARY MATERIALS

Cell lines

786-O was purchased from Cell Lines Service (Eppelheim, Germany) and LNCaP prostatic cancer cell line was obtained from ATCC.

IC50 determination

HT29, HCT116, SW480 and SW620 colon carcinoma cells (10^4 to 2.10^4) were treated with different concentration of VPA (ranging from 0 to 80mM). After two days, cell viability was determined by MTT assay and IC25 and IC50 were calculated.

Measurement of cell number and cell death

Cells (5×10^5) were plated in 6-well plates, untreated or treated with CRAd (MOI 15.6 vp/cell), VPA (low or high dose) or both for 3 days. After incubation with propidium iodide, total cell number was determined using Accuri C6 flow cytometer (BD Biosciences).

Supplementary Table 1: CRC cell line sensitivity to VPA

CRC cell lines	IC50 (mM)	IC25 (mM)
HT29	10	5
HCT116	1.2	0.6
SW480	10	6.2
SW620	5	2.5

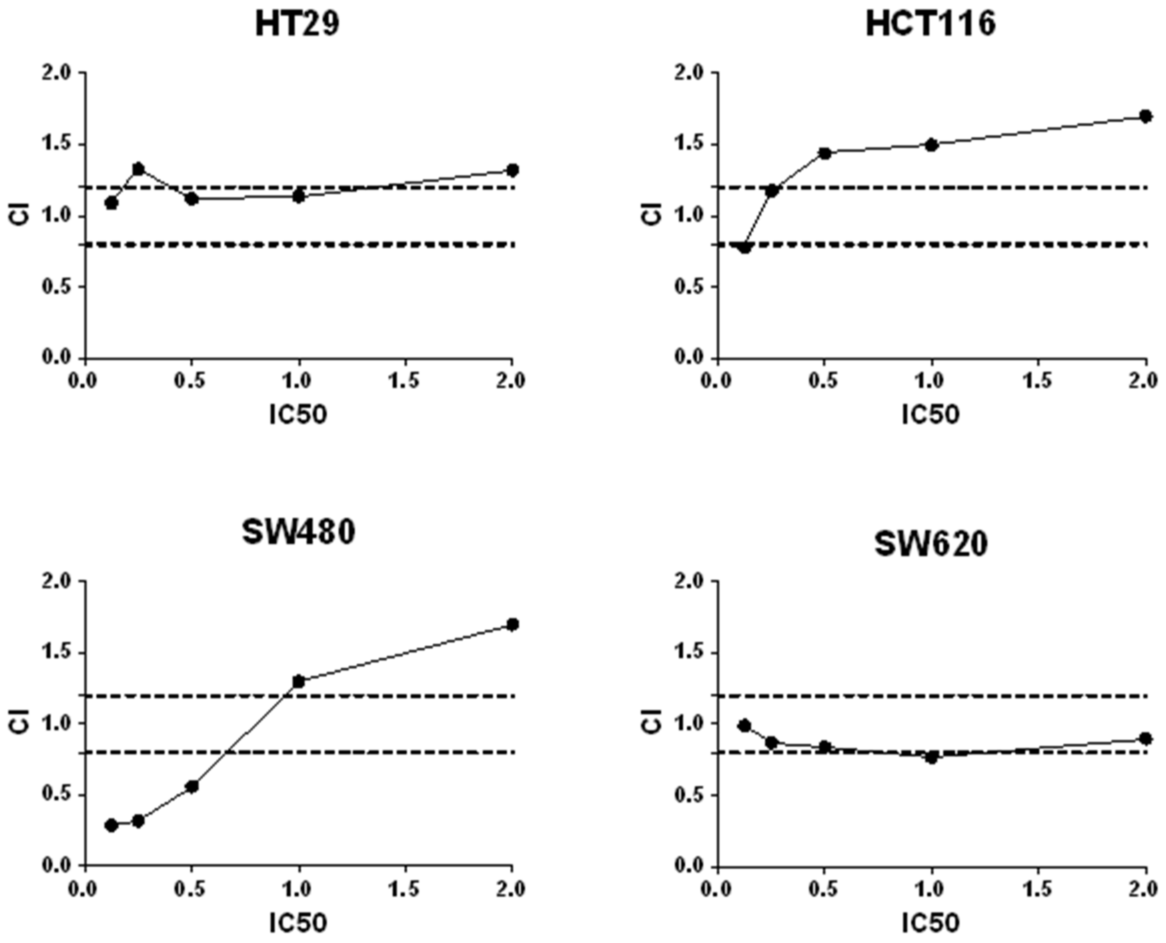

Supplementary Figure 1: Chou-Talalay analysis of interaction between CRAd and VPA. CRC cell lines (HT29, HCT116, SW480 and SW620) (5×10^5) were treated for 3 days with different concentrations of CRAd and VPA. Data of cell survival determined by a MTT assay were calculated as fraction affected and CI were calculated using CompuSyn software. CI < 0.8, synergistic interaction; 0.8 < CI < 1.2, additive interaction; CI > 1.2 antagonistic interaction.

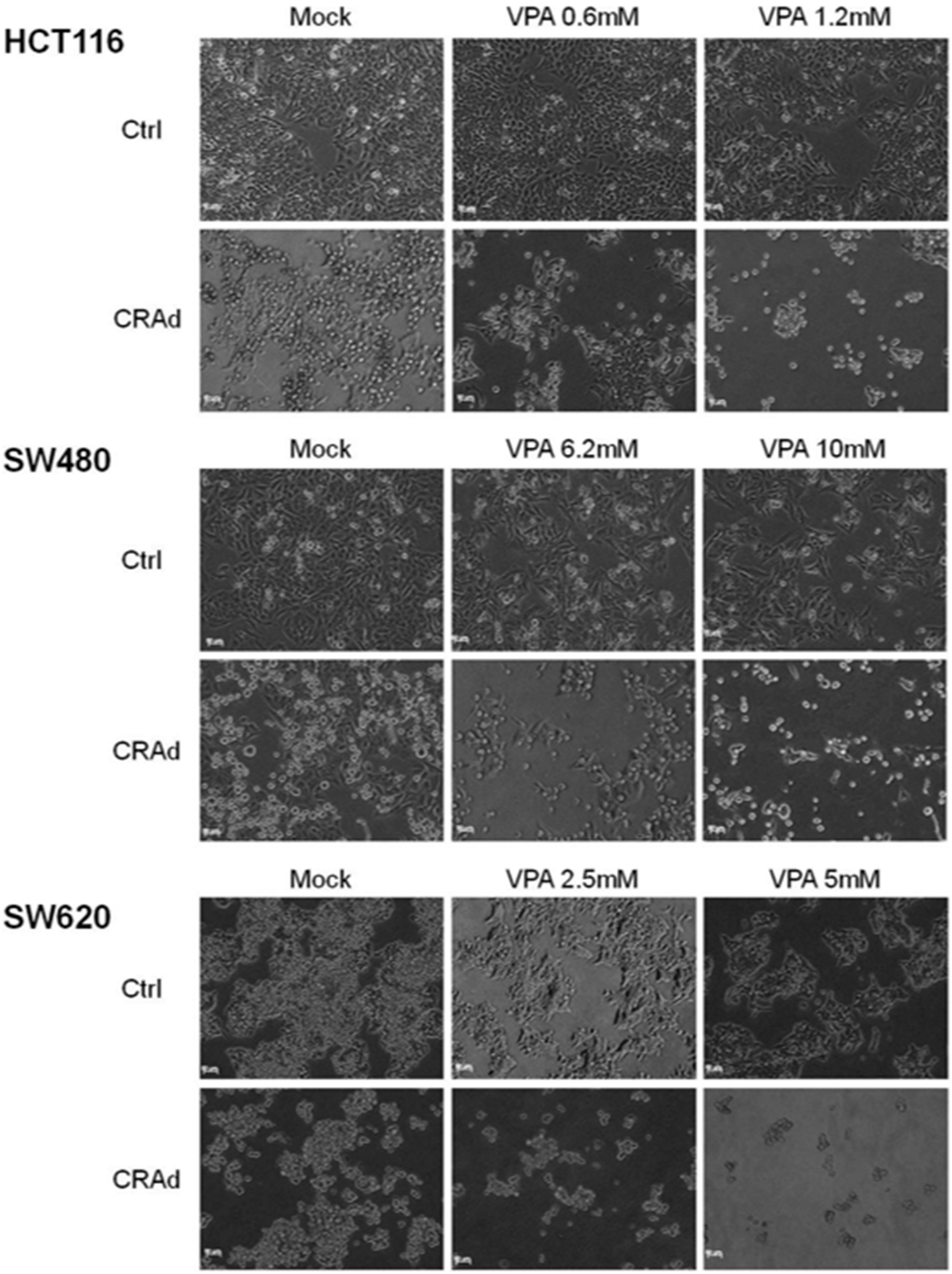

Supplementary Figure 2: Microscopic observation of colon cancer cells after co-treatment with CRAd and VPA. CRC cell lines (HCT116, SW480 and SW620) (5×10^5) were untreated or treated with CRAd (MOI 15.6 vp/cell), VPA (IC25 and IC50) or both. After 3 days, cells were observed by phase-contrast microscopy (scale bar, 50μm).

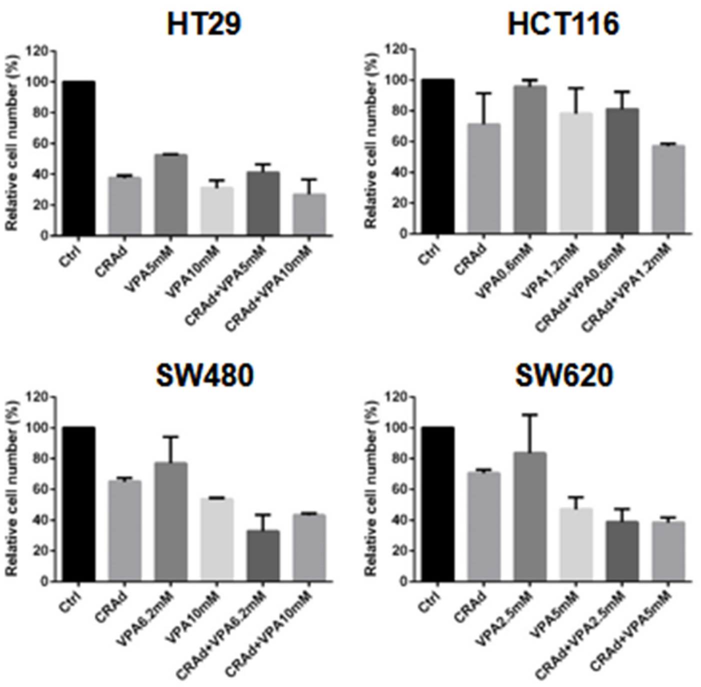

Supplementary Figure 3: Reduction in cell number after co-treatment with CRAd and VPA. CRC cell lines (HT29, HCT116, SW480 and SW620) (5×10^5) were untreated or treated with CRAd (MOI 15.6 vp/cell), VPA (IC25 and IC50), or both. After 3 days, cell number (mean + SD) was determined by flow cytometry and the results were expressed relative to untreated cells.

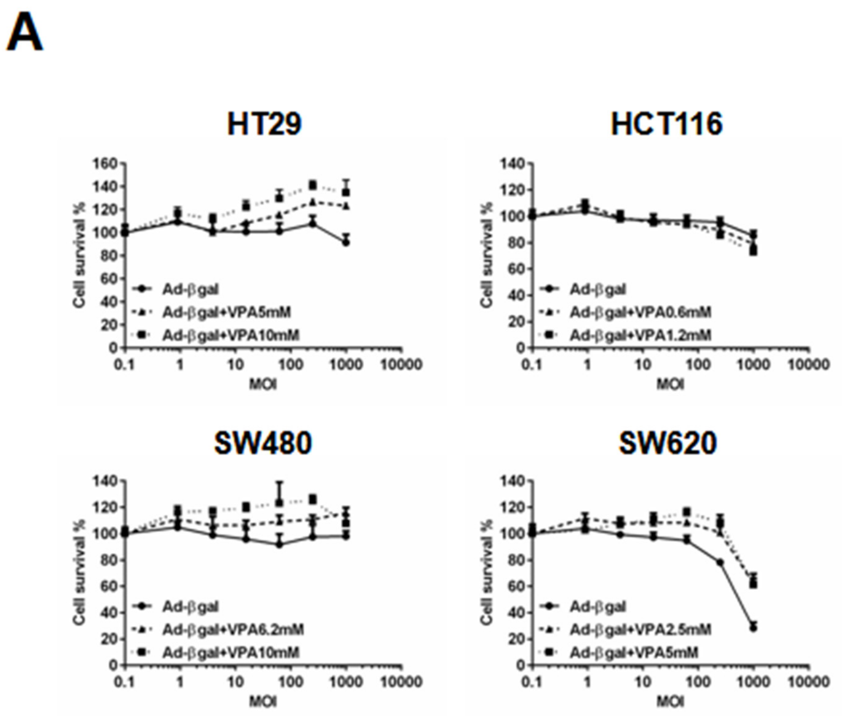

Supplementary Figure 4A: Inability of a replication-deficient adenovirus to reduce the survival of CRC cell lines in the presence of VPA. HT29, HCT16, SW480 and SW620 cells (10^4) were treated with Adβgal (MOI ranging from 0 to 1000 vp/cell), VPA (IC25 and IC50), or both. After 3 days, cell viability was measured with a MTT assay. The results (means + SD) were expressed relative to non-infected cells treated with different VPA doses. The results are representative of two experiments.

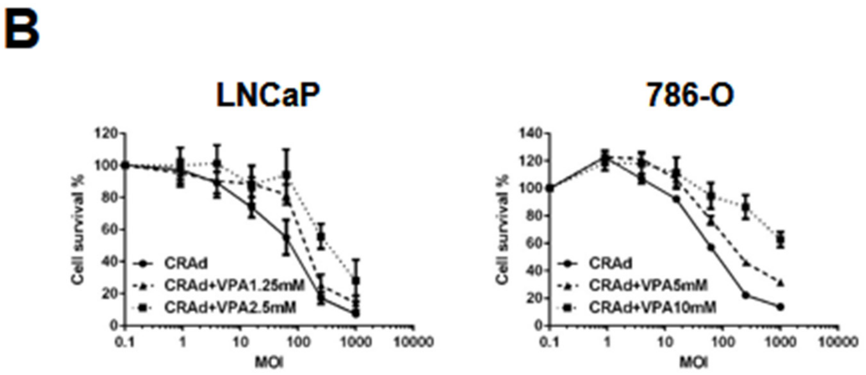

Supplementary Figure 4B: Inability of CRAd to reduce the survival of prostatic and renal carcinoma cells in the presence of VPA. Prostatic (LNCaP) or renal (786-O) carcinoma cells were treated with CRAd (MOI ranging from 0 to 1000 vp/cell), VPA, or both. After 3 days, cell viability was measured by a MTT assay. The results (means +/- SD) were expressed relative to non-infected cells treated with different VPA doses. The results are representative of two experiments.

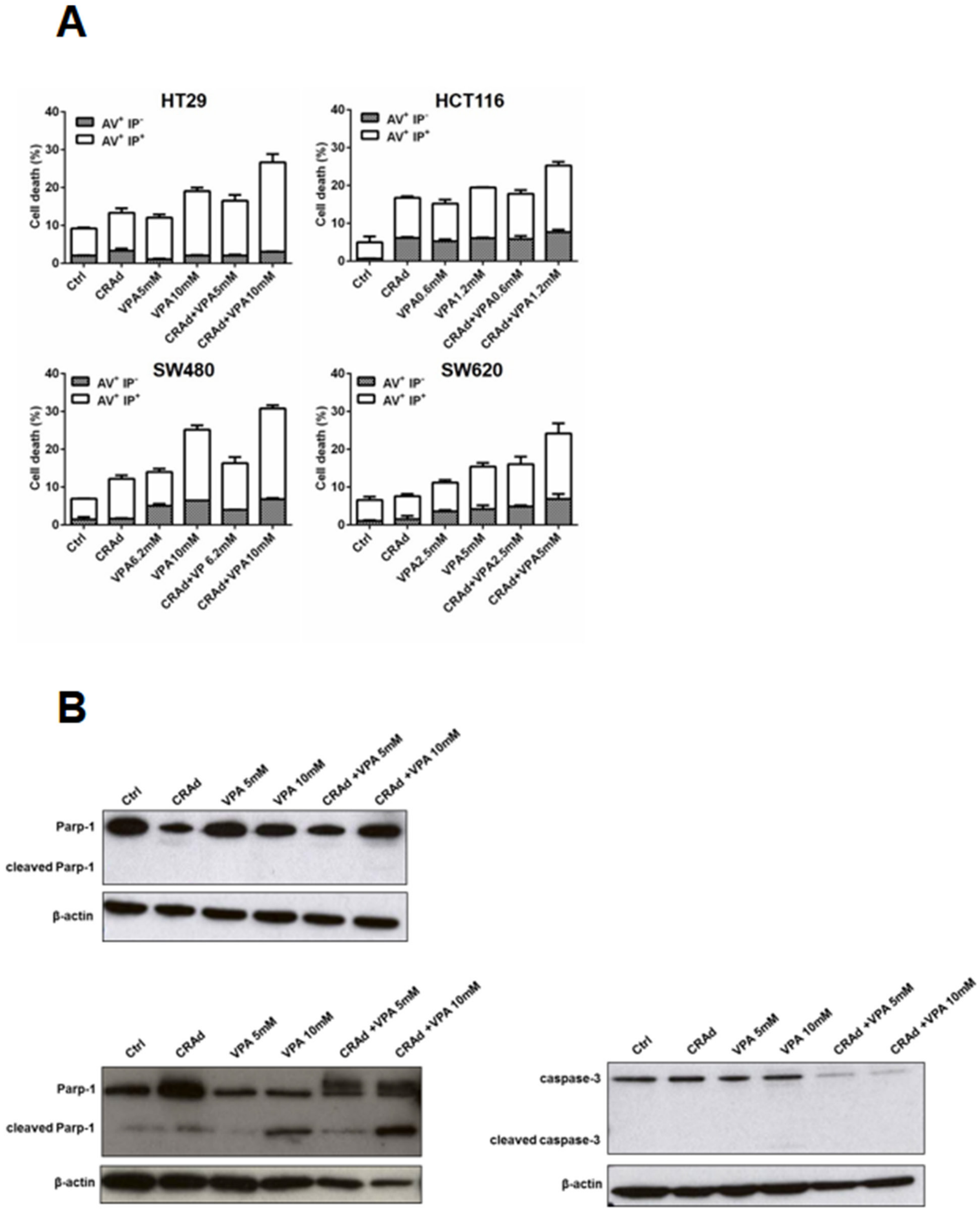

Supplementary Figure 5: Cell death after the co-treatment of CRC cell lines with CRAd and VPA. Colon cancer cells (HT29, HCT116, SW480 and SW620) (5×10^5) were untreated or treated with CRAd (MOI 15.6 vp/cell), VPA (IC25 and IC50), or both. **(A)** After 3 days, annexin V-positive and propidium iodide-positive (AV^+PI^+) and AV^+PI^- cells were quantified (mean + SD). Results are expressed as a percentage of total cells. **(B)** Detection by western blot of Parp-1 (left, upper and down correspond to day 2 and 3 post-treatment, respectively) and caspase-3 (right, day 3 post-treatment) proteins in HT29 cells.

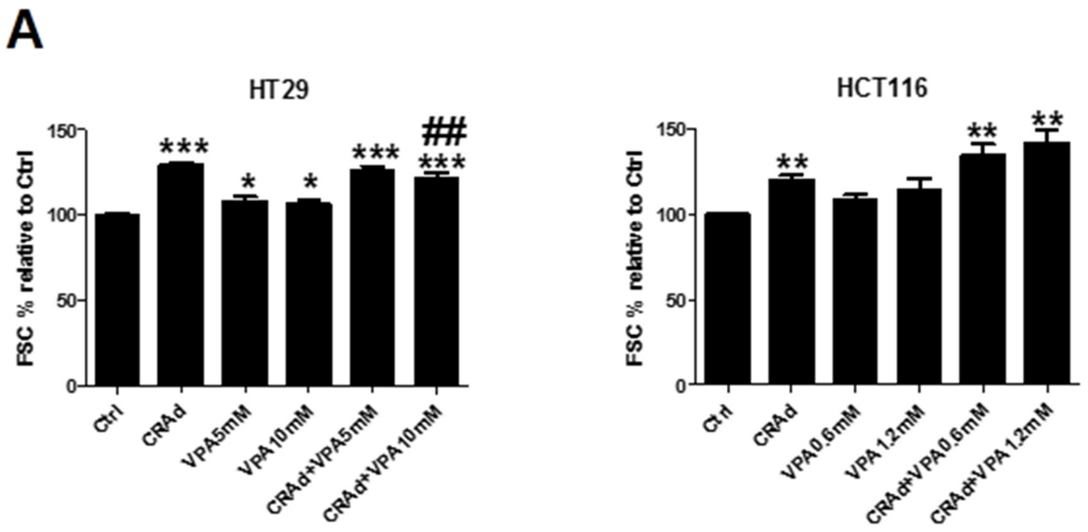

Supplementary Figure 6A: CRAd infection increased cell size. HT29 and HCT116 cells were mock-infected or infected with non-replicative Adβgal (MOI 15.6vp /cell) and/or treated with VPA (IC25 and IC50). After 3 days, cytometry measurement of FSC was used as an indicator of cell size. The results were expressed relative to mock-infected (Ctrl). Means + SEM, data obtained from 5 (HT29) and 3 (HCT116) experiments. * $P < 0.05$, ** $P < 0.01$, *** $P < 0.001$ relative to Ctrl; $^{\#\#}$ $P < 0.01$ relative to CRAd.

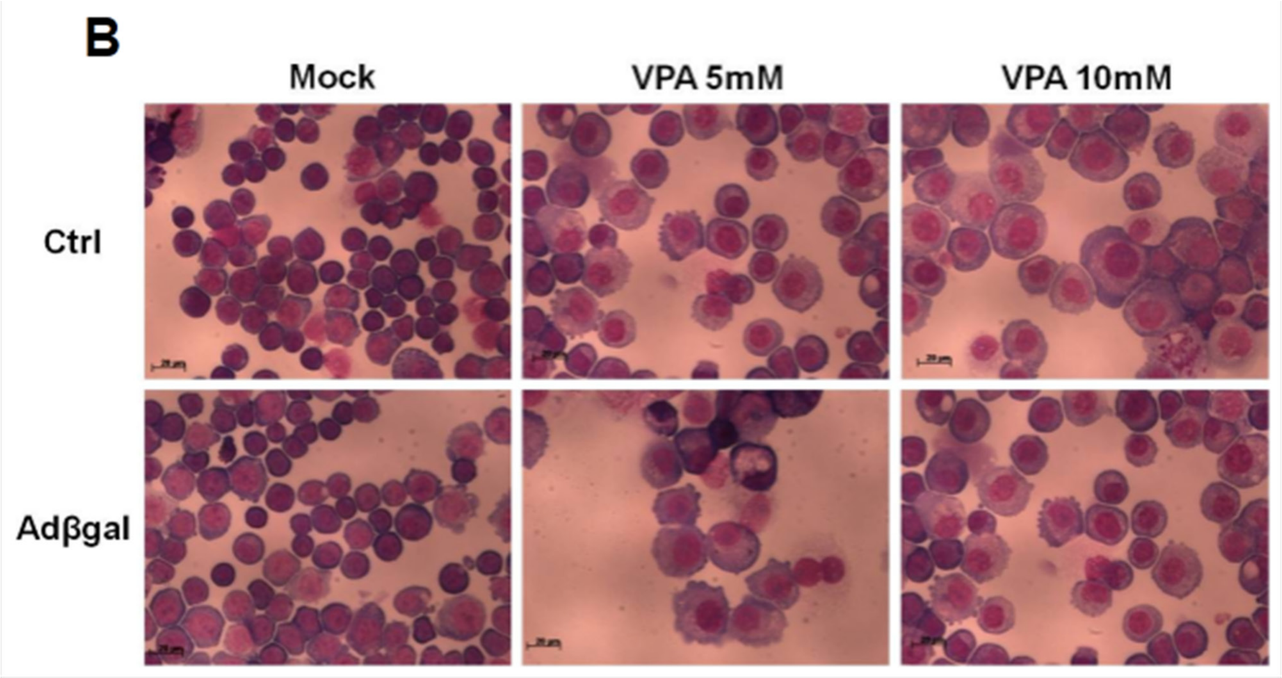

Supplementary Figure 6B: Co-treatment with replication deficient Ad and VPA did not induce polyploidy nor increase in size of HT29 cells. HT29 cells were mock-infected or infected with replication deficient Adβgal (MOI 15.6vp /cell) and treated with VPA (IC25 and IC50). After 3 days, cells were attached by cytospin and stained with Wright-Giemsa. Cell size and nuclei were observed under phase-contrast microscope (scale bar 20µm).

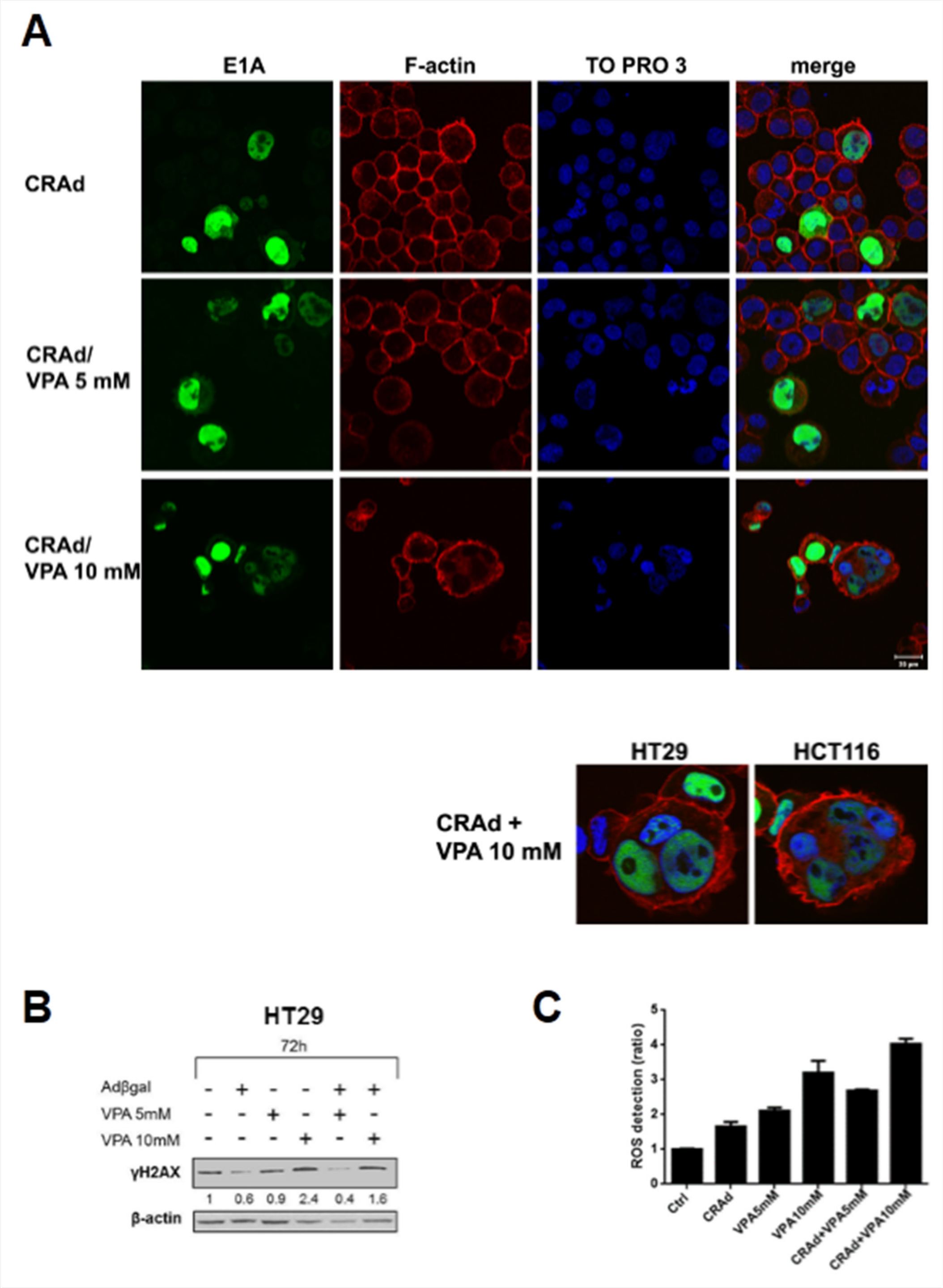

Supplementary Figure 7: Association of viral infection with polyploidy in colon carcinoma cells after treatment with CRAd and VPA. (A) Upper pannel, HCT116 cells were untreated or treated with CRAd (MOI 15.6 vp /cell), VPA (IC25 and IC50), or both. Infected cells (E1A), cell limits (phalloidin) and nuclei (TO-PRO-3) were observed after 2 days by confocal microscopy with merge shown (scale bar 20µm). TO-PRO-3 is represented arbitrary in blue. Lower panel, higher magnification is shown for both HT29 (enlargement of Figure 4D) and HCT116 (see upper panel) in CRAd and VPA condition. **(B)** HT29 cell line was untreated or treated with replication-deficient Adβgal (MOI 15.6vp/cell), VPA (IC25 and IC50), or both. H2AX phosphorylation (γH2AX) was measured by western blot. The numbers indicate the level of γH2AX relative to untreated cells. **(C)** ROS production in HT29 cells after 3 days of the indicated treatment. The results (means + SD) are expressed relative to control cells.

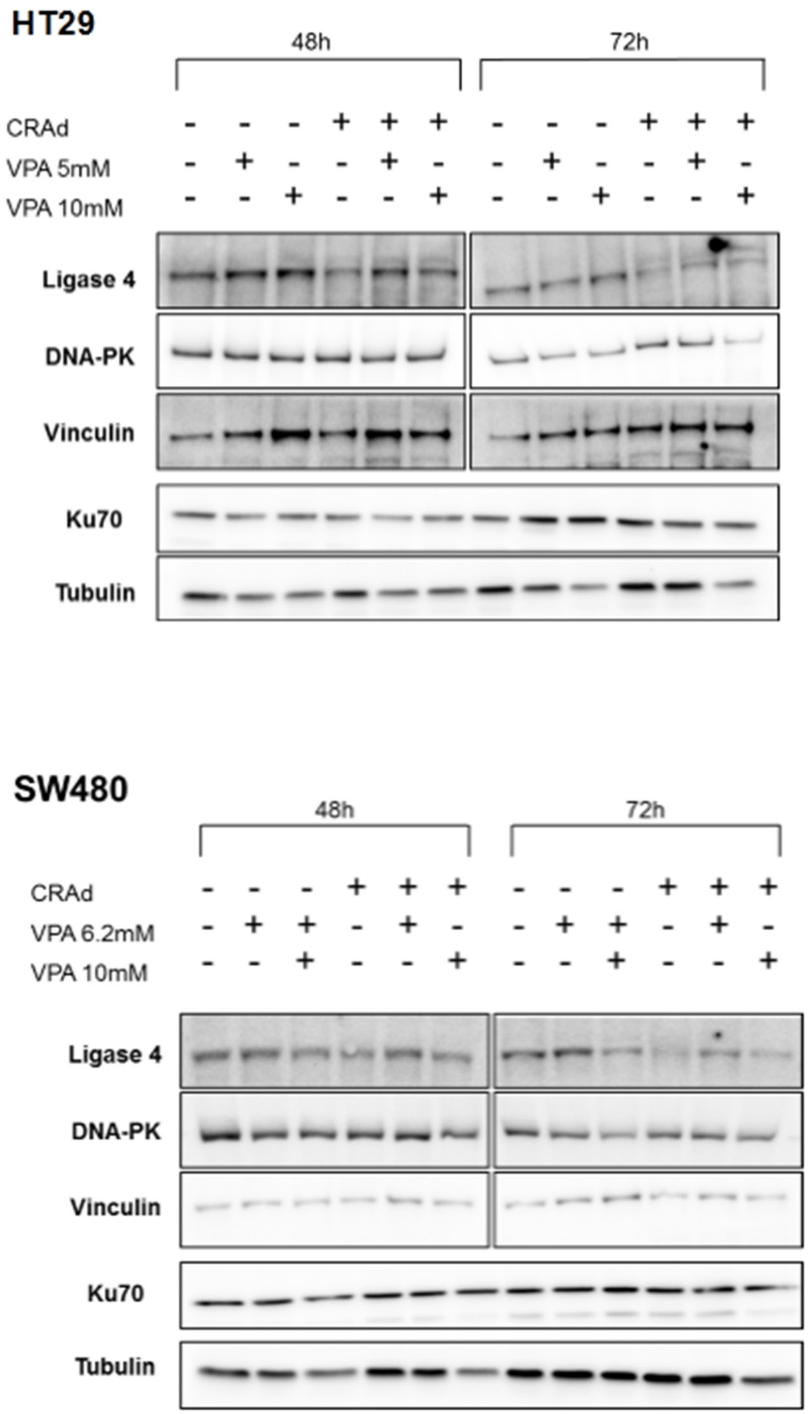

Supplementary Figure 8: CRAd-induced inhibition of ligase IV level in CRC cell lines. HT29 and SW480 cells were untreated (Ctrl) or treated with CRAd (MOI 15.6 vp /cell), VPA (IC25 and IC50), or both. Expression of DNA repair proteins at the indicated time points was measured by western blot. Upper, HT29; lower, SW480.

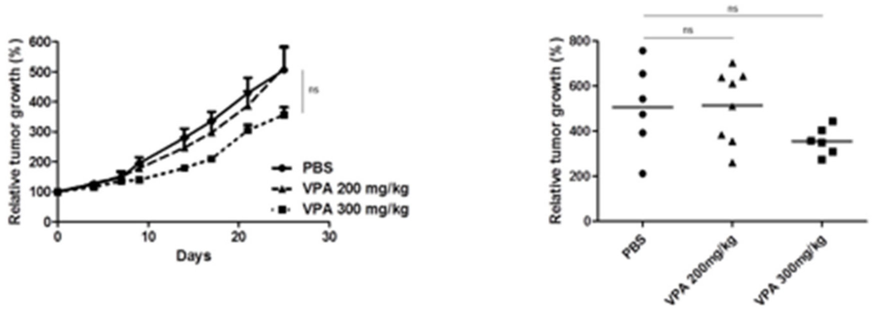

Supplementary Figure 9: Sensitivity of HT29 tumors to VPA. Mice bearing HT29 xenografts were injected intraperitoneally with daily injections of VPA (200 mg/kg or 300 mg/kg) or PBS. Tumor volumes at different time points are expressed relative to tumor volumes at the beginning of treatment. Kinetic of tumor growth showing means + SEM (left) and tumor volumes at day 25 (right) with dots and bars representing results of individual mice (n = 6 to 8) and means, respectively, is shown. ns, non significant.
